# How can big data analytics be used for healthcare organization management? Literary framework and future research from a systematic review

**DOI:** 10.1186/s12913-022-08167-z

**Published:** 2022-06-22

**Authors:** Nicola Cozzoli, Fiorella Pia Salvatore, Nicola Faccilongo, Michele Milone

**Affiliations:** Department of Economics, University of Foggia, Via Caggese n.1, Foggia, Italy

**Keywords:** Healthcare management, Healthcare organization, Healthcare governance, Big data analytics

## Abstract

**Background:**

Multiple attempts aimed at highlighting the relationship between big data analytics and benefits for healthcare organizations have been raised in the literature. The big data impact on health organization management is still not clear due to the relationship’s multi-disciplinary nature. This study aims to answer three research questions: a) What is the state of art of big data analytics adopted by healthcare organizations? b) What about the benefits for both health managers and healthcare organizations? c) What about future directions on big data analytics research in healthcare?

**Methods:**

Through a systematic literature review the impact of big data analytics on healthcare management has been examined. The study aims to map extant literature and present a framework for future scholars to further build on, and executives to be guided by.

**Results:**

The positive relationship between big data analytics and healthcare organization management has emerged. To find out common elements in the studies reviewed, 16 studies have been selected and clustered into 4 research areas: 1) Potentialities of big data analytics. 2) Resource management. 3) Big data analytics and management of health surveillance systems. 4) Big data analytics and technology for healthcare organization.

**Conclusions:**

In conclusion is identified how the big data analytics solutions are considered a milestone for managerial studies applied to healthcare organizations, although scientific research needs to investigate standardization and integration of the devices as well as the protocol in data analysis to improve the performance of the healthcare organization.

**Supplementary Information:**

The online version contains supplementary material available at 10.1186/s12913-022-08167-z.

## Background

Big data is transforming and will transform the healthcare organizations in the near future [1, 2]. Scientific literature in the managerial context applied to healthcare organizations, consider the Big Data Analytics (BDA) a fundamental tool, so much so that it has attracted the attention of the scientific community and stakeholders [3]. However, a premise should be made: data by themselves explain little, thus, to be useful in the healthcare organization management, firstly it is necessary to validate their quality, and secondly, find the right correlations. In other words, the data should be processed, analyzed, and interpreted with the appropriate tools [4, 5].

Technological applications in healthcare BDA-related are rapidly increasing [6] and will increasingly characterize managers’ decision-making process. For example, IBM’s Watson project [7] is a "super-computer" that has scoured through several million scientific articles over the last twenty years and uses artificial intelligence tools (e.g., Machine Learning) to correlate disease symptoms and predict possible diagnostic scenarios. This case helps to understand how and to what extent BDA could really support healthcare managers to improve their decision processes, while increasing the performance of the healthcare organization.

Nowadays, the amount of data is no longer an issue. Internet traffic reports from Cisco and other network operators have estimated the entire digital universe to be 44 zettabytes and 463 exabytes will be the daily information could be generated by 2025. A new era took place in which the processes of production and management of human knowledge will no longer be the exclusive preserve of humans; machines will also play their part as knowledge producers [8]. From pharmaceutical companies to healthcare organizations, this enormous potential of data products, combined with IoT applications and AI tools [9–11], will play a significant role in the near future. Today, the medical applications based on IoT allow the monitoring of clinical data through the production of data generated by special devices (e.g., wearable devices) [12], remotely accessible by a physician rather than by caregivers [13].

The market size is a useful indicator of how much the healthcare organizations are turning their attention to new management models based on the use of big data. By 2025, the big data market in healthcare will touch $70 billion with a record 568% growth in 10 years. The use of such a tool not only represents a complex challenge [14], but also opens opportunities for all those involved in the healthcare supply chain who manage decision-making processes. Moreover, if on the one hand this technology will influence the definition of new managerial strategies within healthcare organizations, on the other hand, it will have positive repercussions on the effectiveness and efficiency of healthcare processes [15]. Indeed, the big data technology is used by healthcare managers to get, for example, information related to the list of doctors and nurses, the list of drugs with their expiration date, etc., in order to have tools for facilitating decision-making processes, improving the quality of services provided, and, at the same time, rationalizing the use of resources, by facilitating the management of the healthcare organization as a whole.

The BDA satisfies multiple needs that, on the one hand, influence the quality of the healthcare organization’s performance and, on the other hand, are useful in directing management strategies to improve the supply of healthcare services. Below there are some strategies, which aim to:

- Provide specific services to patients, from diagnostics to preventive medicine passing through therapeutic adherence.
- Detect the onset and spread of diseases in advance.
- Observe parameters inherent to hospital quality standards, promoting control and prevention actions.
- Modify treatment techniques.
- Facilitate research and development in pharmacology, reducing the time to market of drugs.
- Facilitate research and development of new and specific medical devices.

The main aim of this research is, therefore, to provide both an integrative framework on the state of art, and perspectives on how the BDA can be useful for the management of the healthcare organization. Considering the results, food-for-thought on how this technological and cultural revolution will affect the *modus operandi* of healthcare organizations will be launched.

Through an overview of recent scientific studies, this research aims to raise awareness among both practitioners and managers about BDA tools applied to healthcare management to address more effectively and efficiently the challenges imposed by an increasing demand for healthcare services.

In this regard, the study provides a systematic literature review (SLR) to explore the effect of BDA on the healthcare management by analyzing articles from the Scopus database during a period of 5 years (2016 – 2021).

Furthermore, the result through a content analysis, aspires to be a privileged starting point to find out potential barriers and opportunities provided by BDA-based management systems for smarter healthcare organization. Specifically, the study answers different research questions (RQs) as different levels of analysis have been performed. By analyzing the relationship between BDA-based management systems and the benefits delivered to the organizations, the research could not be conducted without exploring the state of art of BDA tools deployed in the field of healthcare. Thus, starting from this background the discussion on the future perspectives on BDA development in the healthcare organizations appears as a need.

## Theoretical framework

Why use BDA and how to exploit its potential for healthcare organization management? This is the main question asked by managers and decision makers working in the healthcare sector. In recent years there have been multiple attempts in the literature aimed at highlighting the relationship between implementation of BDA and benefits for healthcare organizations, in terms of both resource efficiency and process management.

In 2017, a study by Wang and Hajli [16] has proposed a model founded on Resource-Based Theory and BDA Capabilities (BDAC) to explain the relationship between BDA, benefits, and value creation for healthcare organizations. As stated by Srinivasan and Swink [17], BDAC refers to “*organizational facility with tools, techniques, and processes that enable a firm to process, organize, visualize, and analyze data, thereby producing insights that enable data-driven operational planning, decision-making, and execution*”. In the healthcare organization, BDAC represents the ability to collect, store, analyze, and process huge volume variety, and velocity of health data come from various sources to improve data-driven decisions [18, 19]. Indeed, the study of Wang and Hajli [16], validated on an empirical basis by 109 cases of BDA tools implementation in 63 healthcare organizations, has demonstrated how specific "path-to-value" can be identified. By varying degrees of relevance of the identified pathways, it has been shown that alongside the challenges of implementing certain BDA tools, there are corresponding specific benefits for healthcare organizations. Preliminarily, the study has defined the ability to analyze big data through the concept of Information Lifecycle Management (ILM) [20]. In this perspective, the capabilities of the BDA in healthcare organizations are configured as the abilities to process health data from diverse sources and provide significant information to healthcare managers. Thorough BDA, managers can detect timely indicators and identify business strategies, which allow them to put in place perspective plans, efficient strategies, and programs to increase the performance of organizations.

Researchers have found that BDA capabilities primarily stem from the implementation of various tools and features. Specifically, in order of importance, BDA capabilities are firstly triggered by processing tools (e.g., OLAP, machine learning, NLP), followed by aggregation tools (e.g., data warehouse tools), and, secondly, by data visualization tools and capabilities (e.g., visual dashboards/systems, reporting systems/interfaces).

Among the potentials triggered by the implementation of BDA in the healthcare organization, the analytical one was the main capability, that is the ability to process clinical data characterized by immense volume, variety (from text to graph), and speed (from batch to streaming), using descriptive analysis techniques [21, 22]. In this regard, it is important to note that BDA-based management systems are the only ones capable of analyzing semi-structured or unstructured data. This represents a crucial element for revealing correlation patterns that are difficult to determine with traditional management systems [23]. Furthermore, the launch of these systems in a healthcare organization ensures the ability to effectively manage outputs regarding care process and service in order to constantly improve the performance of the organization. In summary, the characteristics of BDA-based management systems implemented in a healthcare organization, are:

- predictive analytics capability, i.e., the ability to explore data and identify useful correlations, patterns and trends, and extrapolate them to predict what is likely to occur in the future [24, 25];
- interoperability capability, i.e., the ability to integrate data and processes to support management, collaboration, and sharing across different healthcare departments, managers, and facilities [26], and finally,
- traceability capability, i.e., the ability to integrate and track all patient history data from different IT facilities and different healthcare units.

In terms of expected benefits from the BDA implementation, the study of Wang and Hajli [16] has showed that the most important ones are obtained from improved operational activities, such as improved quality and accuracy of healthcare decisions, rapid processing of issues, and the ability to enable treatments proactively before patients’ conditions worsen. Next, in terms of relevance, they were the benefits related to IT infrastructure, such as standardization and reduced costs for redundant infrastructure and the ability to quickly transfer data between different IT systems. Substantially, they have delivered a useful business model that healthcare managers can draw on to evaluate the specific leverages they need to activate in relation to the implementation of the BDA-based management systems. In addition to highlighting the undoubted benefits, the authors clearly show how specific BDA tools can facilitate the decision-making processes of healthcare managers and make them faster and more effective.

In another study carried out to identify BDA benefits and supports, and to drive organizational strategies, Wang, Kung, and Byrd [19], through the analysis of 26 case studies related to the BDA applications in the healthcare organization, have identified five "capabilities" of BDA: analytic capability for care patterns, unstructured data analytical capability, decision support, predictive, and traceability capabilities [19]. The study is remarkably interesting because in addition to mapping precise benefits, it also recommends specific strategies considering the BDA implementation for healthcare organizations. These strategies are useful for achieving effective results by leveraging the potential of BDA.

The first successful strategy is to implement governance based on the use of big data, starting with a definition of objectives, procedures, and key performance indicators (KPIs). Once again, one of the discriminating factors for success in implementing such a strategy remains the integration of information systems and the standardization of data protocols that often come from heterogeneous sources already existing in healthcare organizations. The second strategy is related to developing a culture of data sharing. The third one considers the training of healthcare managers, who cannot ignore knowledge related to BDA, for example on the use of data mining and business intelligence tools. The fourth strategy is related to the storage of big data, often available in heterogeneous formats, and is identified in the transition from the more expensive traditional storage systems (NAS) to more efficient and effective systems such as cloud computing solutions. The last strategic driver involves pathways related to the implementation of predictive BDA models. The mastery of KPIs, interactive visualization and data aggregation tools such as dashboards and reports should be acquired instruments for healthcare managers and in general for healthcare organizations oriented to BDA driven process management strategies.

More recent studies focus attention on the management practices supply chain in healthcare. In the study performed by Yu et al. [27], the authors, interviewing senior executives in Chinese hospitals, show on both a theoretical and empirical basis, how BDAC positively impacts the three dimensions of hospital supply chain integration (SCI) (inter-functional integration, hospital-patient integration and hospital-supplier integration) and how SCI, in turn, contributes to improve the operational flexibility [27]. By “operational flexibility” in the healthcare organization, it is meant the ability of a ward to adapt its operating procedures in relation to unforeseen circumstances while meeting the needs of patients [28, 29].

The scholars have delivered an important contribution in demonstrating the relationship between BDAC, SCI, and operational flexibility from multiple perspectives, by providing useful management guidance for healthcare executives and managers involved in the supply chain. By analyzing and processing medical and managerial data with advanced analytical techniques, Chinese healthcare organizations were able to facilitate decision-making process with timely and appropriate actions, for example, tracking people's movements during the lockdown caused by the Coronavirus, understanding ongoing health trends, and managing pharmaceutical supplies [30, 31].

This theoretical framework provides a key to interpreting the benefits offered by good practices deriving from the use of the BDA in the healthcare organization.

At the same time, the rigorous scientific method allows the validation of empirical experiences in relation to clear theoretical references. In the next paragraph projects that demonstrate what is stated in the literature are shown.

## Practical framework

*N(ursing)* + *Care* App is an mHealth application that supports the work of frontline health workers (FHW) in developing countries [32]. The system is designed to collect not only patient data, but also diagnostic images. It is also given the opportunity to add recommended doctors based on the advice of FHWs in case the patient needs to follow a specific hospital visit.

For healthcare managers, predicting the number of emergency department accesses is a critical issue which complicates the optimization of the human resource management. To this end, Intel, and Assistance Publique-Hôpitaux de Paris (AP-HP), the largest hospital university in Europe, leveraging datasets from multiple sources, worked together to build a cloud-based solution to predict the number of patient visits to emergency rooms and hospital admissions. This predictive analytics tool, will enable healthcare managers at AP-HP hospitals to know the number of emergency room visits and hospital admissions at 15 days in order to reduce wait times, optimize human resource (HR) levels based on anticipated needs, accurately plan patient loads, including by pathology, and overall improve the quality and efficiency of services provided by the healthcare organization [33].

Chronic conditions, if not kept under control through a rigorous program of therapeutic adherence, can become a source of both more serious physical problems for patients and economic burdens for healthcare organizations. Another project that actively introduced BDA tools into healthcare management was carried out by the European Commission to launch production of the drug *Enerzair Breezhaler*. It was the first drug for the treatment of asthma co-packaged and co-prescribed with the Propeller digital platform. The app sends a reminder to comply with therapeutic adherence and maintains a record of the data, which the patient shares with him or her physician. Studies have demonstrated that the Propeller platform increases the degree of asthma control by up to 63%, therapeutic adherence by up to 58% [34], and reduces asthma emergency department visits and hospital admissions by up to 57% [35].

The practical framework described, aided by some empirical experience, only partially reveals the potential offered by BDA. The diffusion of BDA-based management systems in the healthcare organization will trigger a virtuous circle, allowing soon to accumulate increasingly accurate medical data. By exploiting the most advanced AI technologies, BDA will support predictive analysis, allow physicians to make more accurate and faster diagnostic pathways and managers to use results. It will help health practitioners in the decision-making process, optimize the use of resources with a consequent costs reduction and, overall, improve the quality of services provided by healthcare organizations.

## Methods

The main aim of this study is to update the state of art about the BDA-based management systems adopted in the healthcare organization, underlining management advantages for both the organizations and managers. BDA has the potential to reduce the cost of care, prevent disease outbreaks, and improve the patients’ quality of life. Through its ability to process and cross-reference massive amounts of both management, and clinical information, BDA promises to be an effective support tool for both healthcare managers and patients.

To achieve this aim, a Systematic Literature Review (SLR) was performed. This method identifies, evaluates, and summarizes the updates that raise from the literature about the BDA tools used to improve both the healthcare organizations performance and patients’ quality of life. The method takes inspiration from the protocol used by Khanra S., et al. [36] which considers inclusion and exclusion criteria.

The present study aims to add a contribute to the literature by addressing three RQs:

- What is the state of art of BDA adopted by healthcare organizations?
- What about the benefits for both health managers and healthcare organization?
- What about future directions on BDA research in healthcare?

**Fig. 1:**
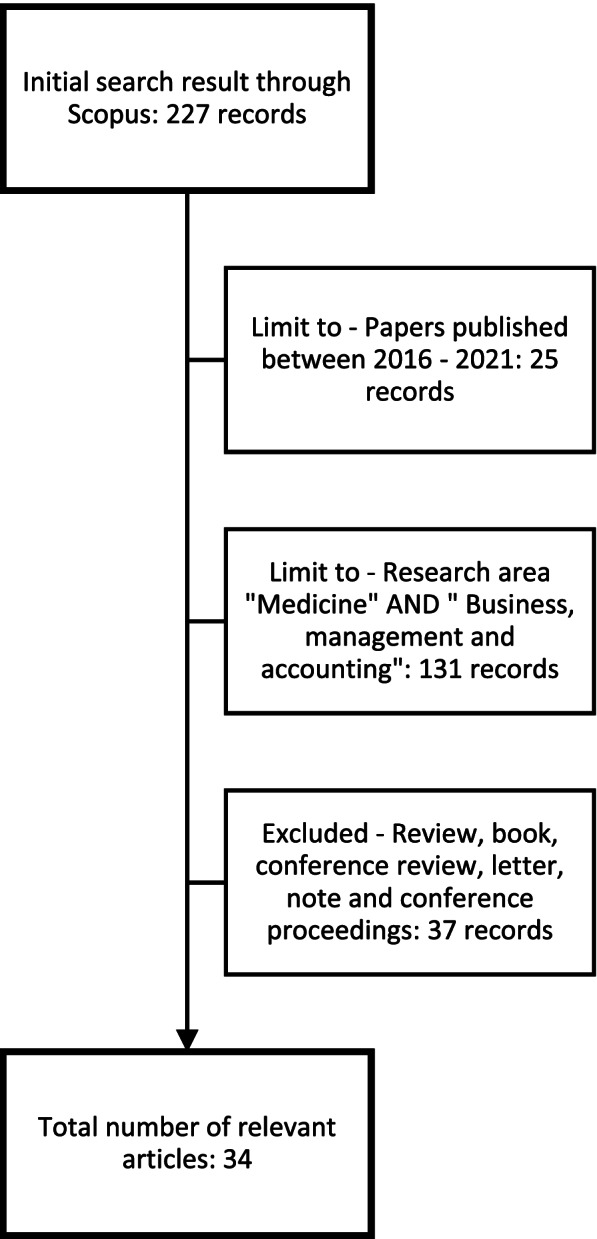
Workflow of articles selection

To answer the RQs, as widespread electronic database Scopus has been selected. To obtain an international validity of studies, the research only considers papers in English. Utilizing the Boolean operator “AND”, the following keywords have been searched: “big data analytics” AND “healthcare” AND “management”. As inclusion criteria, only papers published from 2016 to 2021 have been considered. As subject areas, “medicine” and “business, management and accounting” have been selected. Instead, as exclusion criteria, article in press and the following documents type: “review”, “book”, “conference review”, “letter” and “note” have not been taken into account. Also, to avoid a dispersal of the study, conference proceedings have been excluded. Following the searching protocol, 34 results have been obtained (Fig. 1).

An excel spreadsheet was used to perform the extraction procedures while the statistical analyses were carried out using the software STATA 16 ©. The list of the extracted papers investigated with the content analysis can be found in the Appendix.

The work proceeds through a descriptive analysis. After that, a content analysis has been performed to identify the most relevant characteristics of the BDA-based management systems, underlining the positive impact for the healthcare organizations, without neglecting to outline the trends for the future scenarios and research directions.

## Results

According to the SLR, the iterative process shown in the Fig. 1, has allowed to delete the duplicates and match the results with the RQs.

As shown in Fig. 1 the initial search on Scopus database has delivered 227 results. By limiting research to papers published between 2016 and 2021, 11% of records have been removed. At the second stage, by selecting the subject areas, the screening has allowed to exclude 131 records; thus, the 57.7% of the results initially selected. The last step of the process has conducted to exclude document types such as Review, Book, Conference Review, Letter, and Note. In other words, 37 records were excluded, representing 16.3% of the sample. At the end of the screening process, 34 articles were selected, representing about 15% of the sample.

**Fig. 2:**
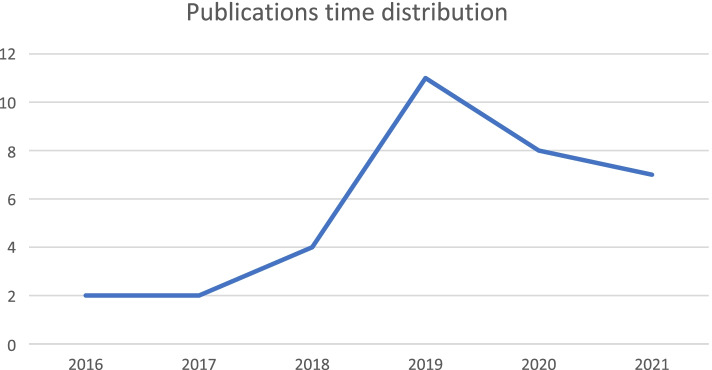
Trend of research steams

In the descriptive analysis the time distribution of the studies from 2016 to 2021 is included. It is important to note the increasing of publication trend from 2017 to 2019. This output confirms a growing interest in the research field of BDA applied to healthcare organizations (Fig. 2).

The trend of research steams considers a sample of 34 scientific contributions as they come from the screening process above described. Although 6% of the total sample was collected in the years 2016 and 2017, it is only indicative of the growing trend of scientific studies on BDA in healthcare sector. The overall incidence in 2018 was 12% but the turning point was reached in 2019 as 32% of the studies collected in the sample were reached. This outcome could be read considering the Covid-19 pandemic outbreak which has been a representative testing ground for BDA tools by helping managers and decision-makers to plan healthcare managerial strategies.

In this context, the use of the BDA by Chinese healthcare organizations for tracking people's flow during the lockdown, represents an important case study that has registered the peak in the time flow of research. By looking at 2020 and 2021 data, which represent respectively 24% and 21% of the total scientific contributions, the growing trend seems to be confirmed by validating the rising interest in BDA research seen as a planning tool for healthcare processes.

The pie-chart shows the scientific production by country. It is necessary to specify that Scopus database clusters the studies by home country author’s organization, therefore the same study could be referred to more than one country and thus belong to more than one cluster.

**Fig. 3:**
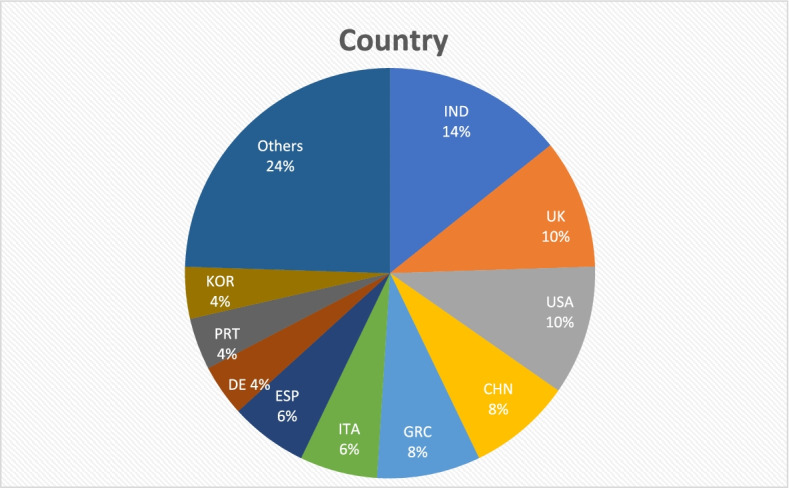
Geographical locations of the studies

The geographical locations of the studies showed in the Fig. 3 outlining India, UK, and USA as more than one third of the total scientific producers. It is well known that IT companies as Google, Apple, Amazon, and Microsoft are investing considerable resources on BDA tools for healthcare. China and India contribute together with 22% of the scientific articles. Big data technology has played a key role in virus tracking during the pandemic crisis. The "Internet Plus Healthcare", a big data center in Zhongwei (China), provides cloud services to both healthcare institutions and IT companies. In Yinchuan (China), an industrial park for big data acts as a catalyst for IT company involved in healthcare sector. India confirms to be one of the heavily adopter countries of artificial intelligence, big data analytics, and IoT technologies. Although India must face the challenge to provide basic healthcare services in a predominantly rural country, start-ups with BDA skills in healthcare are springing up.

It is also important underlining the performance of the European countries. UK, Greece, Italy, Spain, Germany, and Portugal support the research with almost 40% of the studies published, confirming that Europe will be a driving force for the BDA research in the next future. The development of a European Health Data Space (EHDS) is an ambitious project of the European Commission. It will lead member states to share an efficient infrastructure for both exchange and management health data by providing citizens with equal treatment, free access to clinical data, and quality healthcare services.

In the area “Others” all the other countries contributing marginally to research have been included.

The next step of the study is focused on a content analysis to show the experiences of applying BDA in healthcare organizations.

Starting from the 34 articles selected for the descriptive analysis, to identify in detail the core issue of the study, a second screening was performed. 18 articles were excluded because weakly focused on the research objective which concerns specifically how BDA can be used for healthcare organization management. Thus, after an in-depth reading of abstracts and full papers, the scholars have identified 16 papers closer targeted on the mentioned research objective. The 16 studies selected through a content analysis were clustered into 4 research areas (RAs) as showed in the following table (Table 1). The clustering procedure identifies 4 relevant topics: Potentialities of BDA (RA1), Resource management (RA2), BDA and management of health surveillance system (RA3), BDA technology for healthcare organization (RA4). The proposed clustering has been though to give an easy-to-go research map and to support the healthcare managers.

**Table 1:** Clusters by relevant topics

Cluster	Title	Authors	Years	N° of papers per cluster
RA1 – Potentialities of BDA	Exploring the path to big data analytics success in healthcare	Wang, Y. and Hajli, N	2017	4
	Role of big data analytics capability in developing integrated hospital supply chains and operational flexibility: An organizational information processing theory perspective	Yu, W. et al	2021	
	Decision-Making based on Big Data Analytics for People Management in Healthcare Organizations	Sousa, M.J., et al	2019	
	Integrated care and connected health approaches leveraging personalized health through big data analytics	Maglaveras, N. et al	2016	
RA2 – Resource management	Big data-enabled solutions framework to overcoming the barriers to circular economy initiatives in healthcare sector	Kazançoğlu, Y. et al	2021	5
	The impact of big data analytics and artificial intelligence on green supply chain process integration and hospital environmental performance	Benzidia, S. et al	2021	
	Evidence-based Public Health Policy Models Development and Evaluation using Big Data Analytics and Web Technologies	Moutselos, K. and Maglogiannis, I	2020	
	The Role of Big Data and Twitter Data Analytics in Healthcare Supply Chain Management	Alotaibi, S. et al	2020	
	A survey on big data analytics in medical and healthcare using cloud computing	Kundella, S. and Gobinath, R	2019	
RA3 –BDA and management of health surveillance system	An assessment of factors that affect the implementation of big data analytics in the Zambian health sector for strategic planning and predictive analysis: A case of Copperbelt province	Chellah, R.C. and Kunda, D	2020	3
	Benefits and challenges of Big Data in healthcare: an overview of the European initiatives	Pastorino, R. et al	2019	
	Setting up a regional health system database for seamless population health management in Singapore. Proceedings of Singapore Healthcare	Gunapal, P.P.G.et al	2016	
RA4 – BDA technology for healthcare organization	Big Data in home healthcare: A new frontier in personalized medicine. Medical emergency services and prediction of hypertension risks	Clim, A. et al	2019	4
	The Challenges of Diagnostic Imaging in the Era of Big Data	Aiello, M. et al	2019	
	A survey on big data management in health care using IOT	Bharathi, M.J. and Rajavarman, V.N	2019	
	Big Data and Machine Learning: A Way to Improve Outcomes in Population Health Management. (Book Chapter)	Martinez, F.E.L., et al	2018	

### RA1: potentialities of BDA

Wang and Hajli [16] define BDA potentialities in the healthcare context as “*the ability to acquire, store, process and analyze large amounts of health data in various forms, and deliver meaningful information to users, which allows them to discover business values and insights in a timely fashion*”. The relationship between BDA and the benefits for the healthcare organizations it has been well expressed by the theory of the “path to value chain” [16]. This path represents an important contribution to the exploration of business value, not only for drawing the generic and well-established connection between big data capabilities [19] and the benefits, but also for empirically showing how capabilities can be developed and what benefits can be achieved in the healthcare organizations. Another study included in this area, explores the key role of BDA capabilities in developing healthcare supply chain integrations and its impact on hospital flexibility [27]. Specifically, the BDA has a fundamental role in developing healthcare integration supply chain and the operational flexibility. Considering the health and economic crises caused by the Covid-19, this dimension of BDA has been an especially important leverage for managers to improve operational flexibility of the healthcare organizations. The ability to provide predictive models and real-time insights, is a powerful prospective of the BDA for helping healthcare professionals and managers in decision-making process. In this regard, the literature presents several applications of big data in healthcare that support the data collection, management, and integration of data in healthcare organizations [37]. Moreover, BDA enables the integration of massive datasets, supporting decisions of manager and monitoring the managerial aspects of healthcare organizations. Building a decision-making process based on BDA, firstly means identifying the big data keys that can implement *ad-hoc* strategies to improve efficiency along the healthcare value chain. To this end, the research carried out by Sousa et al., [37] underlines the benefits that BDA can give to the decision-making process, through predictive models and real-time analytics, assisting in the collection, management, and integration of data in healthcare organizations.

To date, thanks to an integrated and interconnected ecosystem, is becoming possible to provide personalized healthcare services, collect an enormous quantity of both clinical and biometrics data and, thus, implement BDA instruments. Nevertheless, to take a real advantage from these tools and turn them into useful decision support systems (DSS), is necessary for R&D to be focused on data filtering mechanisms in order to obtain good-quality reliable information [38]. The healthcare models based on BDA and implementation of new healthcare programs, enable both medical and managerial decision support for the healthcare services provision. New types of interactions with and among users of the healthcare ecosystem will produce in the next future a wide variety of complex data, thus, the main challenges refer to information processing and analytics.

In light of the above, the RA1 includes studies for which the quality of data and the need for high performance filtering mechanisms are becoming keys factor for the success of BDA-based management systems in the healthcare organizations. For example, the study carried out by Maglaveras et al., [38], included in this area, explores new R&D pathways in biomedical information processing and management, as well as to the design of new intelligent decision support systems.

### RA2: resource management

Another important research direction emerged from the literature review, concerns positive impact of the BDA on the resource management. Insufficient policy for managing medical materials waste, energy use and environmental burden, restricts the resources conservation. The BDA is extremely useful in this aspect; it could provide in the next future an important contribution to implement the circular economy processes and to support sustainable development initiatives in the healthcare organizations [39]. To this end, the study developed by Kazançoğlu et al. [39], underline the importance of circularity and sustainability concepts to mitigate the sector’s negative impacts on the environment. Furthermore, the study identifies the barriers related to circular economy in the healthcare organization and provides solutions to these barriers by implementing BDA-based management systems. Lastly, the authors, have developed a managerial, policy and theoretical framework to support healthcare managers to launch sustainable initiatives in the context of healthcare organization.

The impact on the performance has been also investigated by studies that have linked benefits of BDA and artificial intelligence with green supply chain integration process [40]. Digital learning is more becoming a “moderator” of the green supply chain process with a significant positive impact on environmental performance of the healthcare organization. BDA-AI technologies will lead to improvement of the environmental process integration and green supply chain collaboration and, consequently, will support the managers’ decisions involved in the supply processes. This study also provides an important reference framework for logistics/supply chain managers who want to implement BDA-AI technologies for supporting green supply processes and enhancing environmental performance of the healthcare organization [40].

Nowadays, many scholars are focusing on BDA-driven decision support systems to sustain the healthcare managers [41]. These types of BDA-based analytical tools will provide a useful quantitative support for managers of healthcare organizations. The authors have reported design and technical details of the system implementations using case studies. They have developed a toolkit which represents a framework reference for resources management, allowing to create strategic models and obtain analytical results for evidence-based decisions and managerial evaluations.

In this RA, two other important topics investigated by BDA are: high quality healthcare service, and healthcare costs. Optimize the supply chain activities is an imperative to keep lower the healthcare costs. The data generated by medical equipment and devices can be successfully used in forecasting, decision-making process, and to make more efficient the healthcare supply chain management [42]. The study carried out by Alotaibi et al. [42], thus, presents a review on the use of big data in healthcare organizations underling opportunities and challenges deriving from the application of BDA-based management systems within the organizations.

As already asserted, a good implementation of BDA in the healthcare organization will play a fundamental role in improving the clinical outcomes management, giving helpful insights for decision makers and managers, in order to avoiding diseases, reducing healthcare expenses, and improving the performance of the healthcare organization [43]. However, to achieve these ambitious outcomes the research will face a crucial challenge: how to rationalize, make easily usable, and at affordable costs, heterogeneous data coming from diverse sources. The research developed by Kundella and Gobinath [43] represents an important contribute to explore key challenges, techniques, technologies, privacy issues, security algorithms and future directions of the use of BDA in the healthcare organization.

### RA3: BDA and management of health surveillance system

The rise of BDA promises to solve many healthcare challenges in the developing countries. The BDA applied to healthcare organization help managers to rationalize the resources, and health system to better delivery treatments to the patients [44]. In this regard, the government of Zambia is thinking to implement BDA solutions to provide more effective and efficient healthcare services. A well-managed health surveillance system represents an important driver to improve the quality of life and reduce the medical waste, especially in developing countries where the lack of resources is severe and limits economic development. For all these reasons, Europe is investing on BDA initiatives in public health and in the oncology sectors, to generate new knowledge, improve clinical care and make more efficient the management of the public health surveillance system [45]. The BDA capability for identifying specific population pattern, managing high volume of data and turn it into real (or near real) time insights, contributes to identify it as a powerful tool to support the managers for the decision-making processes. Despite this, implementing a BDA-based management systems within the healthcare organizations requires investment in the human capital, strong collaboration with stakeholders, and data integration with and among the healthcare units. To this end, Gunapal et al., [46] has highlighted that Singapore has setup a Regional Health System (RHS) database to facilitate BDA for proactive population health management (PHM) and health services research [46]. The structure of the healthcare database has been built collecting data from four database coming from three RHSs: National Healthcare Group (NHG), Tan Tock Seng Hospital (TTSH), National University Hospital (NUH) and Alexandra Hospital (AH). The result has been a database including information useful for the healthcare managers which incorporates data on patient demographics, chronic disease, and healthcare utilization information. These characteristics facilitate the identification of specific patients’ paths linked by past healthcare utilization and chronic disease information. Converging information into a single database helps to understand the cross-utilization of healthcare services across the three RHSs. A such approach allows to setup the RHSs structure for initiative-taking population health management (PHM) and to improve the performance of healthcare organizations [46].

### RA 4: BDA technology for healthcare organization

The wearable devices and different kind of sensors, able to collect clinical data, in combination with BDA, will constitute the basis of personalized medicine and will be crucial tools to improve the performance of healthcare organizations [47]. The scientific research has to face the important challenge to adapt data acquisition, storage, transmission and analytics to healthcare demand. Thus, the healthcare data should be categorized, homogenized, and implemented into specific models by adapting machine-learning techniques to the nature of the healthcare organization.

A fruitful field of interest for the application of BDA in healthcare organization is the diagnostic imaging. To take out maximum benefits from it and to be useful for managers of healthcare organizations, it is necessary to implement digital platforms and applications [48]. Indeed, the simple production of a large amount of data does not automatically translate to an advantage for the healthcare performance. Specific applications are required to favor the correct and advantageous management of diagnostic images [48]. The link between BDA and IoT technologies, as instrument to incorporate the accessibility, capacity to customize, and practical conveyance of clinical data, emerged as another research direction investigated by the papers included in this RA. These tools allow: (1) the healthcare organizations to decrease expenses; (2) the people to self regulates treatments; (3) practitioners to take as quickly as possible decisions in remote way and keep constant contact with patients [49].

In light of these results, it is possible to state that IoT, big data, and artificial intelligence as machine-learning algorithms, are three of the most significative innovations in the healthcare organization. These types of organizations are implementing home-centric data collection networks and intelligent BDA systems based on machine learning technologies. For example, a high-level implementation of these systems has been efficiently implemented in Cartagena, Colombia, for hypertensive patients by using an e-Health sensor and Amazon Web Services components [50]. The authors stress the importance of using the combination of IoT, big data, and artificial intelligence as tools to obtain better health outcomes for the communities and improved performance for healthcare organization. The new generation of machine-learning algorithms can use standardized data sets generated by these sources to improve the effectiveness of public health interventions [50]. To this end, as pointed out by numerous studies in the field of BDA applied on healthcare organizations, it becomes crucial for the next future research to concentrate R&D efforts towards full standardized dataset protocols.

## Discussion

As highlighted by the results, in Europe, as well as in the rest of the world, a significant trend is emerging among healthcare organizations in adopting BDA-based management systems [45]. Among the clustering process performed, the common element in the studies reviewed is the positive relationship between BDA tools and achievable benefits for healthcare organizations.

As emerged by the RAs, some studies explore business value for healthcare organizations and the concept of potentialities of BDA (RA1) to explain the evidence of precise path-to-value chains leading to specific benefits [16]. These perspectives provide useful guidelines for healthcare managers who want to consider implementing BDA tools in their organizations. Some authors in particular focus on the role of BDA capabilities in the development of hospital supply chain integration and operational flexibility, demonstrating a positive relationship between the two dimensions [27]. During the Covid-19 outbreak, it became clearer how important operational flexibility is to healthcare organizations. The scholars also underline how BDA can impact to the efficiency of the decision-making processes in healthcare organizations, through predictive models and real-time analytics, helping health professionals in the collection, management, and analysis [37].

In general, BDA-based management systems make personalized care programs possible. However, considering the enormous amount and heterogeneity of information available nowadays, it emerges the necessity to address R&D pathways towards data filtering mechanisms and engineering new intelligent decision support systems within the healthcare organizations [38].

Circular economy (CE) and sustainability concepts are becoming important key drivers in healthcare organizations to reduce negative impact on the environment (RA2). Some study directions look at BDA as tool to provide solution for barriers related to CE and support sustainable development initiatives in the healthcare organizations [39]. Empirical studies have demonstrated the benefits of BDA-AI in the supply chain integration process and its impact on environmental performance. By assessing a sample of 168 French hospitals, Benzidia et al. [40], has observed that the use of BDA-AI technologies has a significant impact on environmental process integration and green supply chain. In particular, this study provides important insights for healthcare managers, who wish to implement BDA-AI technologies for sustaining green supply processes and improving environmental performance [40]. BDA and web technologies can successfully help managers to redesign healthcare processes making them more effective and efficient. Since healthcare spending is constantly growing in the world’s major regions, there is urgent need to redesign processes optimizing supply chain activities such that high-quality services could be provided at lower costs [42]. Although BDA-based management systems promise to fulfil this role in the healthcare organization, more in-depth studies are required. Due to heterogeneity of information sources, one of future research direction should deeply investigate the protocol standardization and integration in data analyzing as well as techniques and technologies used, security algorithms of BDA in the healthcare and medical data [43].

In developing countries, as well as in the rest of the world, the management of health surveillance is a sensitive issue (RA3). Therefore, authors have studied main key factors that hind BDA access in the healthcare organization [44]. Technology, staff, data management and health policies have been identified as some of decisive variables [44]. Due to increasing of the ageing population and the related disability, healthcare organizations will face hard challenges soon. To this end, big data can also help healthcare managers to detect patterns and to turn high volumes of data into usable knowledges. In this context investments in technological infrastructures are needed as well as in the human capital [45]. China is proving, with a large scale of investment, to be a pioneer country in the adoption of BDA-based management systems in the healthcare organization [46].

The rising of AI, IoT, machine learning [49–51], and sensors technology, as well as embedded systems able to communicate each other, have boosted the adoption of BDA with valuable benefits for the healthcare organization (RA4). These technologies will play a fundamental role on big data management to improve the performances of the healthcare organizations. Some authors have underlined privacy issues related to healthcare data and the necessity to make sensor data homogeneous and tagged. Furthermore, implementation of clinical records into models and adaptation of machine-learning techniques is required [47]. Future R&D in this field should be focused on the developing of digital platforms and specific applications based on BDA also for managing diagnostic images [48].

## Conclusions

By exploring the relationship between BDA-based management systems and the benefits delivered to the healthcare organizations, this study replies to 3 RQs: 1) What is the state of art of BDA adopted by healthcare organizations, 2) What are the benefits for both health managers and healthcare organizations and 3) What are the future directions on BDA research in healthcare.

To answer the RQs the SLR has started from an investigation on the recent literature BDA about the BDA in healthcare organizations. Descriptive analysis has been performed on a sample of 34 studies coming from all over the world. The second stage shows a detailed content analysis on 16 studies which better answer to research question about the relationship between benefits for the healthcare organization and BDA solutions.

By analyzing the successful BDA strategies in healthcare context, some authors focus their attention on the BDA potentialities applied in the healthcare organizations [16, 37]. Indeed, the research highlights how analytical tools through personal health systems support public health management systems and how BDA suggests new pathways to support healthcare managers in decision-making process.

In the literature, other scholars highlight the positive impact of BDA on resource management. The BDA solutions are analyzed as tools to sustain CE initiatives [38, 39] as well as to enable green supply chain process integration and improve hospital performance [40]. By exploiting KPIs coming from BDA solutions, some researchers present innovative models for planning public health policy [41]. In this context, the studies consider BDA cloud computing solutions and social media data analytics for supporting the performance of healthcare supply chain management [42, 43]. Furthermore, researchers from all around the world are showing particular interest on BDA for health surveillance system management [44–46].

According to the recent literature, BDA is transforming the healthcare organizations. The SLR has showed how the BDA solutions are now quite considered a milestone for managerial studies applied to healthcare organizations. The Coronavirus pandemic has been a good test run for using BDA to design healthcare policy strategies. Although an extensive literature on BDA to support healthcare management is being produced, the classification into four RAs proposed is an attempt to examine precise key research directions. About that, the limitations of the present research can be detected as the difficulty to review a field of literature constantly evolving. To date, the amount of data is no longer an issue. To be useful in the healthcare context, is necessary to validate their quality and then find the right correlations. In other words, the data should be processed, analyzed, and interpreted correctly. For this reason, emerges the need to address research pathways towards filtering mechanisms, by converting data from big to smart, and engineering new decision support systems within the healthcare organizations [38].

The content analysis carried out in this research has shown that studies are addressed to find out new models for both predictive and personalized medicine by exploiting BDA technologies [47]. The researchers underline the added value of using BDA both in the medical diagnostic process [48] and jointly with IT technologies such as IOT and machine learning [49, 51].

Thus, considering the results obtained, it is possible to state that BDA can effectively help healthcare managers to detect common patterns and turn high volumes of data into usable knowledges. Investments on human capital become a priority to exploit the potential of BDA [45].

To achieve these objectives the future research should provide usable insights and standardized procedures for training healthcare managers and practitioners. AI, machines learning, as well as management strategies, will also play their part as knowledge producers in the healthcare organization. Privacy issues related to healthcare data and also the necessity to make sensor data homogeneous, are becoming crucial research topics to be faced. Finally, due to the heterogeneity of information sources, the future direction of research should investigate the standardization and integration of the protocol in data analysis, as well as the techniques useful for the managerial sector to implement increasingly BDA-based management systems in future healthcare organizations [43].

Nowadays the challenge for healthcare organizations is the development of useful applications BDA-based. According with the circular economy view, the future research directions should be addressed considering the relationship between digitalization and management resources consumption. The data centralization combined with a BDA approach can effectively support circular economy processes in healthcare supply chain by reducing waste and resource consumptions.

Exploiting the BDA’s capabilities will also be a key factor in forecasting and monitoring outbreaks. Future studies will need to focus on developing more efficient models for sharing data in order to improve the performance of healthcare organizations around the world.

## Supplementary Information

**Additional file 1.** List of articles.

## Data Availability

The datasets analyzed during the current study are not publicly available due to data relating to scientific journal names and authors but are available from the corresponding author on reasonable request.
