# Supplementary material for: How can big data analytics be used for healthcare organization management? Literary framework and future research from a systematic review
Source: BMC Health Serv Res. 2022 Jun 22;22:809. doi: 10.1186/s12913-022-08167-z (PMC9213639; doi:10.1186/s12913-022-08167-z)
Supplement: Supplementary file 1 — Additional file 1. List of articles. [file 12913_2022_8167_MOESM1_ESM.docx]

**Appendix**

**List of articles**

| Publication Year | Title | Authors |
| --- | --- | --- |
| 2021 | Big data-enabled solutions framework to overcoming the barriers to circular economy initiatives in healthcare sector | Kazancoglu Y., Sagnak M., Lafci C., Luthra S., Kumar A., Tacoglu C. |
| 2021 | The impact of big data analytics and artificial intelligence on green supply chain process integration and hospital environmental performance | Benzidia S., Makaoui N., Bentahar O. |
| 2021 | The synergic relationship between industry 4.0 and lean management: best practices from the literature | Santos B.P., Enrique D.V., Maciel V.B.P., Lima T.M., Charrua-Santos F., Walczak R. |
| 2021 | Value that matters intellectual capital and big data to assess performance in healthcare. An empirical analysis on the European context | Gravili G., Manta F., Cristofaro C.L., Reina R., Toma P. |
| 2021 | Role of big data analytics capability in developing integrated hospital supply chains and operational flexibility: An organizational information processing theory perspective | Yu W., Zhao G., Liu Q., Song Y. |
| 2021 | COVID-19: a pandemic challenging healthcare systems | Wang L., Alexander C.A. |
| 2021 | New age approaches to predictive healthcare using in silico drug design and internet of things (IoT) | Gupta P.K., Nawaz M.H., Mishra S.S., Parappa K., Silla A., Hanumegowda R. |
| 2020 | A review of the literature on big data analytics in healthcare | Galetsi P., Katsaliaki K. |
| 2020 | Big data analysis techniques to address polypharmacy in patients - A scoping review | Wilfling D., Hinz A., Steinhauser J. |
| 2020 | Big data strategies for government, society and policymaking | Lee J.W. |
| 2020 | Evidence-based Public Health Policy Models Development and Evaluation using Big Data Analytics and Web Technologies | Moutselos K., Maglogiannis I. |
| 2020 | An assessment of factors that affect the implementation of big data analytics in the Zambian health sector for strategic planning and predictive analysis: A case of Copperbelt province | Chellah R.C., Kunda D. |
| 2020 | Anonymity in EU health law: Not an alternative to information governance | Mourby M. |
| 2020 | The role of big data and twitter data analytics in healthcare supply chain management | Alotaibi S., Mehmood R., Katib I. |
| 2020 | Ophthatome™: an integrated knowledgebase of ophthalmic diseases for translating vision research into the clinic | Raj P., Tejwani S., Sudha D., Muthu Narayanan B., Thangapandi C., Das S., Somasekar J., Mangalapudi S., Kumar D., Pindipappanahalli N., Shetty R., Ghosh A., Kumaramanickavel G., Chaudhuri A., Soumittra N. |
| 2019 | Benefits and challenges of Big Data in healthcare: An overview of the European initiatives | Pastorino R., De Vito C., Migliara G., Glocker K., Binenbaum I., Ricciardi W., Boccia S. |
| 2019 | A survey on big data analytics in medical and healthcare using cloud computing | Kundella S., Gobinath R. |
| 2019 | Decision-Making based on Big Data Analytics for People Management in Healthcare Organizations | Sousa M.J., Pesqueira A.M., Lemos C., Sousa M., Rocha A. |
| 2019 | Big Data in home healthcare: A new frontier in personalized medicine. Medical emergency services and prediction of hypertension risks | Clim A., Zota R.D., Tinica G. |
| 2019 | Leveraging fog computing for a secure and smart healthcare | Divya Preetha A., Pradeep Kumar T.S. |
| 2019 | Prediction of the increase in health services demand based on the analysis of reasons of calls received by a customer relationship management | Ramos M.I., Cubillas J.J., Jurado J.M., Lopez W., Feito F.R., Quero M., Gonzalez J.M. |
| 2019 | Real-World Evidence Gathering in Oncology: The Need for a Biomedical Big Data Insight-Providing Federated Network | Geldof T., Huys I., Van Dyck W. |
| 2019 | The challenges of diagnostic imaging in the era of big data | Aiello M., Cavaliere C., D'Albore A., Salvatore M. |
| 2019 | A survey on big data management in health care using IOT | Bharathi M.J., Rajavarman V.N. |
| 2019 | Big data in healthcare: A survey | Farooqi M.M., Shah M.A., Wahid A., Akhunzada A., Khan F., ul Amin N., Ali I. |
| 2019 | A Hadoop/MapReduce based platform for supporting health big data analytics | Kuo A., Chrimes D., Qin P., Zamani H. |
| 2018 | Emergence of Big Data Research in Operations Management, Information Systems, and Healthcare: Past Contributions and Future Roadmap | Guha S., Kumar S. |
| 2018 | Profiling Arthritis Pain with a Decision Tree | Hung M., Bounsanga J., Liu F., Voss M.W. |
| 2018 | Big data and machine learning: A way to improve outcomes in population health management | Martinez F.E.L., Nunez-Valdez E.R. |
| 2018 | Validation of the ICU-DaMa tool for automatically extracting variables for minimum dataset and quality indicators: The importance of data quality assessment | Sirgo G., Esteban F., Gomez J., Moreno G., Rodriguez A., Blanch L., Guardiola J.J., Gracia R., De Haro L., Bodi M. |
| 2017 | Handling Data Skew in MapReduce Cluster by Using Partition Tuning | Gao Y., Zhou Y., Zhou B., Shi L., Zhang J. |
| 2017 | Exploring the path to big data analytics success in healthcare | Wang Y., Hajli N. |
| 2016 | Integrated care and connected health approaches leveraging personalised health through big data analytics | Maglaveras N., Vassiis K., Koutkias V., Chouvarda I. |
| 2016 | Setting up a regional health system database for seamless population health management in Singapore | Gunapal P.P.G., Kannapiran P., Teow K.L., Zhu Z., You A.X., Saxena N., Singh V., Tham L., Choo P.W.J., Chong P.-N., Sim J.H.J., Wong J.E.L., Ong B.K.C., Soh E.F., Foo H.J., Heng B.H. |
